# Process Development of a Liquid-Gated Graphene Field-Effect Transistor Gas Sensor for Applications in Smart Agriculture

**DOI:** 10.3390/s24196376

**Published:** 2024-10-01

**Authors:** Jian Lu, Naoki Shiraishi, Ryo Imaizumi, Lan Zhang, Mutsumi Kimura

**Affiliations:** 1 National Institute of Advanced Industrial Science and Technology (AIST), 1-2-1 Namiki, Tsukuba 305-8564, Ibaraki, Japan; chou-ran@aist.go.jp; 2 National Agriculture and Food Research Organization (NARO), 3-1-1 Kannondai, Tsukuba 305-8517, Ibaraki, Japan; naoki_shiraishi@affrc.go.jp; 3 Division of Chemistry and Materials, Faculty of Textile Science and Technology, Shinshu University, 3-15-1 Tokida, Ueda 386-8567, Nagano, Japan; 23fs403f@shinshu-u.ac.jp (R.I.); mkimura@shinshu-u.ac.jp (M.K.)

**Keywords:** ionic liquid, multi-channel graphene FET, fabrication process, hydrophobic layer, nitrate nitrogen, smart agriculture

## Abstract

A compact, multi-channel ionic liquid-gated graphene field-effect transistor (FET) has been proposed and developed in our work for on-field continuous monitoring of nitrate nitrogen and other nitrogen fertilizers to achieve sustainable and efficient farming practices in agriculture. However, fabricating graphene FETs with easy filling of ionic liquids, minimal graphene defects, and high process yields remains challenging, given the sensitivity of these devices to processing conditions and environmental factors. In this work, two approaches for the fabrication of our graphene FETs were presented, evaluated, and compared for high yields and easy filling of ionic liquids. The process difficulties, major obstacles, and improvements are discussed herein in detail. Both devices, those fabricated using a 3 μm-thick CYTOP^®^ layer for position restriction and volume control of the ionic liquid and those using a ~20 nm-thick photosensitive hydrophobic layer for the same purpose, exhibited typical FET characteristics and were applicable to various application environments. The research findings and experiences presented in this paper will provide important references to related societies for the design, fabrication, and application of liquid-gated graphene FETs.

## 1. Introduction

On-field continuous monitoring of nitrate nitrogen and other nitrogen fertilizers of soil through advanced Internet of Things (IoT) technologies enables the optimization of crop growth, resource efficiency, and cost management by data-driven decision-making processes [1,2,3]. It is also essentially important to environmental stewardship in precision and smart agriculture, as the overuse of nitrogen mineral fertilizers and the release of their residuals into the environment have led to groundwater pollution, stratospheric ozone depletion, and global warming over the past several decades [4].

Among the recently presented technologies, Md. Azahar Ali et al. proposed an all-solid-state miniature potentiometric soil sensor with parts-per-million (ppm) resolution, but the degradation of the Ag/AgCl reference electrode may limit its long-term deployment over months [5]. Mohammed A.E. et al. presented an ion-selective electrode (ISE) electrochemical soil nitrate sensor that utilizes electrochemical impedance spectroscopy (EIS) for direct real-time continuous soil nitrate measurement in the range from 8 ppm to 512 ppm without any soil pretreatment. However, the large size of the sensor probe posed integration challenges with IoT-oriented agricultural implements [6]. Many other technologies have been reported as well, but up to today, none of them have moved into practical applications within distributed sensor networks in the IoT era.

Graphene, a hexagonal lattice of carbon-containing atom-thick layer that possesses remarkable electronic conductivity and thermal characteristics, is believed to be very promising for the detection of gaseous molecules due to its high sensitivity to electric charges in its vicinity and its extremely high surface-to-volume ratio [7,8,9]. Graphene field-effect transistors (FETs) have been reported for detecting various biomolecules and gas molecules [10,11], making them a prominent research topic for precision and smart agriculture applications in recent years. Most graphene FETs employ solid-state gate materials that exhibit low sensitivity and selectivity to a certain molecular gas, necessitating high operating voltages in the range of several tens of volts [12]. It is difficult to monitor soil fertilizers continuously through IoT technologies due to the challenges of both power supply and system integration in a distributed wireless sensor network since the lifetime of the sensor strongly depends on battery life. Additionally, as summarized and pointed out by Raj A. et al., harvesting techniques are largely insufficient for powering IoT nodes due to limited power densities or inconsistencies as to when power is harvested [13].

Inaba A. et al. presented a graphene field-effect transistor gated by ionic liquid for ammonia gas sensing. They demonstrated the operation of the device at an ultra-low voltage of less than 1 V, by which the calculated detection limit was 130 ppb [14]. In this sensor, the graphene channel is chemically modified by the ionic liquid, which selectively absorbs gas molecules. When a gate voltage (V_g_) is applied to the ionic liquid, a non-conductive electric double layer forms near the graphene-FET channel. This results in a field-effect transistor with the ionic liquid as the gate. The absorbed target gas molecules by the ionic liquid are diffused to the surface of the graphene channel, resulting in the change of the drain-source current (I_ds_). Consequently, the device’s sensitivity and selectivity are strongly dependent on the volume and type of the ionic liquid due to its diffusion speed, diffusion distances, and selectivity. Thus, a dedicated structure is needed to ensure its reliability for volume control in practical applications. The detailed device sensing mechanism can be found in Inaba A. et al. [15].

In this study, a compact, multi-channel ionic liquid-gated graphene field-effect transistor (FET) has been proposed and developed for on-field continuous monitoring of nitrate nitrogen and other nitrogen fertilizers in smart agriculture [16]. Nitrate nitrogen, which is easily soluble in water and exists in the soil solution, has a direct effect on crop growth. Our sensor may enable the measurement of nitrate nitrogen concentration from the water vapor emitted from the soil, which is extremely important for on-field continuous monitoring of soil conditions to improve agricultural practices [17]. In this device, a hydrophobic ‘ring’-shaped pattern was designed around the graphene channel. An ultra-small droplet of ionic liquids, i.e., [PMIM][BF4], with controlled volume, was filled onto the surface of the graphene channel to selectively absorb target gas molecules and then change the drain-source current (I_ds_) at different gate voltage (V_g_). Compared to the solid-state graphene FETs, the proposed ionic liquid-gated graphene FETs offer the unique advantages of high sensitivity by ionic liquids with low-voltage operation to the atom-thick nature of graphene [15,17,18]. It is believed to be promising for advancing and transforming future agriculture toward achieving sustainable and efficient farming practices. Compared to the electrolyte-gate GFET developed by P. D. Cabral et al. [19] and the graphene field-effect transistor gated by ionic liquid developed by Inaba A. et al. [14], our device exhibits excellent capability in controlling the volume and location of ionic liquids, making it suitable for sensing trace gas molecules with high resolution. Moreover, ionic liquid-gated graphene FETs are believed to excel in response time over solid electrolyte-gated FETs, which have high electrolyte resistance, quicker absorption and dis-absorption, and faster diffusion speed. However, developing a robust device structure with scalable and sensor-compatible fabrication processes that allow for easy filling of ultrasmall volumes of ionic liquids, minimal graphene defects, and high process yields is still necessary for commercial viability.

In this paper, CYTOP^®^ (CTL-809M, Asahi Glass Co., Ltd., Tokyo, Japan) and a photosensitive hydrophobic layer (LDW-N010, Tokyo Ohka Kogyo Co., Ltd., Kawasaki, Japan) were used to create the hydrophobic ‘ring’-shaped pattern. Correspondingly, two different processes using the CYTOP^®^ and the photosensitive hydrophobic layer were developed, evaluated, and compared for the fabrication of our proposed devices. A 6-channel ionic liquid-gated graphene FET with typical FET characteristics was successfully obtained on a single chip to investigate the dependency of graphene channel size on response time, the selectivity of the ionic liquid for various gases, and the sensitivity of the device at different gas concentrations.

This paper demonstrates that the use of the ultra-thin photosensitive hydrophobic layer enables the successful filling of ionic liquids with volumes of 100 nL or less, while the CYTOP^®^ allows for larger volumes of up to 600 nL but at the cost of reduced yields due to chemical residues etc. The process difficulties, major obstacles, and improvements are discussed in detail. Additionally, the fabricated devices were evaluated using a self-developed soil simulation system to confirm their functionality as a gas sensor after the filling of the ionic liquids.

## 2. Experimental

### 2.1. Device Layout and Structure

**Figure 1:**
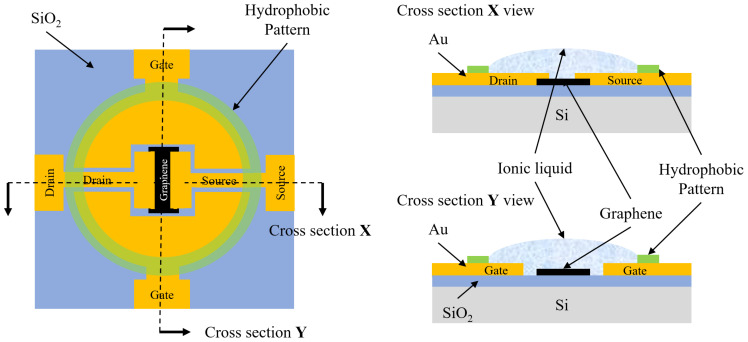
Schematic view of the structure and the layout of each ionic liquid-gated graphene FET (left figure), and the cross-section views of the graphene channel in X direction (upper-right figure) and in Y direction (lower-right figure).

The detailed structure, layout, and cross-section views of each ionic liquid-gated graphene FET are shown in Figure 1. A CVD-grown single graphene layer was used to create the graphene-FET channel. The SiO_2_ on the backside of the silicon was removed to enable the applications when back gates are needed.

To investigate the selectivity of the ionic liquid for various gases, a hydrophobic ‘ring’-shaped pattern was designed around each graphene channel, as shown in the right panel of Figure 1. This pattern restricts the position and controls the volume of the ionic liquid during the subsequent filling process. A thin layer of Au/Cr with a thickness of 36 nm/4 nm was deposited on each side of the graphene channel to serve as the source and drain electrodes. The gate electrode was designed on both sides of the graphene channel for better contact with ionic liquids and easy assembly.

To study the dependency of the channel size on response time, we designed six ionic liquid-gated FETs with different channel sizes on the same chip. The channel length was designed to be 20 μm and 100 μm. The channel width was designed to be 50 μm, 100 μm, 250 μm, and 500 μm. The size of the chip with six channels of ionic liquid-gated graphene FETs was 5 mm × 5 mm.

### 2.2. Fabrication Process Using CYTOP^®^

The device fabrication started by using a 4-inch silicon wafer with a 100 nm-thick SiO_2_ passivation layer on the top surface of the wafer. Since graphene is unsuitable to grow directly on SiO_2_/Si substrate, a commercially available CVD-grown single graphene layer, sized 50 mm × 50 mm, was transferred from a copper foil onto the 4-inch SiO_2_/Si wafer using a wet process. Raman spectroscopy was used to ensure fewer defects and contamination during the graphene transfer [20].

**Figure 2:**
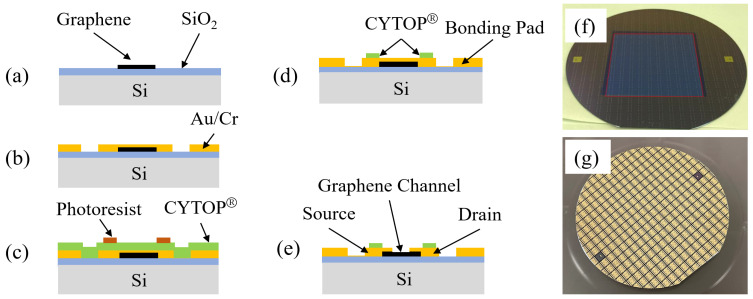
Fabrication process flow using CYTOP^®^: (**a**) graphene etching by O_2_ plasma; (**b**) Au/Cr electrode deposition by sputter and patterning by lift-off; (**c**) CYTOP^®^ coating and (**d**) etching; (**e**) channel release by Au/Cr wet etching. (**f**,**g**) are photos of the wafer after graphene etching in (**a**) and after channel release in (**e**), correspondingly.

CYTOP^®^ is a fluoropolymer that dissolves in exclusive fluorine-based solvents, making it suitable as a thin film coating with excellent hydrophobic properties. Figure 2 shows a detailed process flow of CYTOP^®^ being used to create a hydrophobic ‘ring’-shaped pattern surrounding the graphene channels to facilitate the easy deposition and filling of the ionic liquids. Firstly, as shown in Figure 2a,f, the graphene layer was patterned using oxygen plasma reactive ion etching (RIE), followed by the Au/Cr electrode layer deposition via sputter and patterning by a lift-off process, as shown in Figure 2b. Then, the hydrophobic CYTOP^®^ layer was deposited by spin-coating, baked at 80 °C for 30 min and 200 °C for 30 min, and patterned by oxygen plasma RIE as shown in Figure 2c,d. Finally, as shown in Figure 2e, the graphene channel was released by removing Au/Cr on the top of the channel via wet etching.

The dry etching of a graphene single layer has been reported by P. D. Cabral et al. [19], and the detailed process for CYTOP^®^ coating, baking, and RIE etching has been published elsewhere [21,22]. The photoresist was used as the etching mask for oxygen plasma RIE in both the graphene etching and CYTOP^®^ patterning. Before coating the photoresist on CYTOP^®^, oxygen plasma RIE was applied to the surface of CYTOP^®^ for 10 s to enhance the adhesive force between the photoresist and the CYTOP^®^.

A two-step process was used in the oxygen plasma RIE of CYTOP^®^. The process parameters were as follows: RF power 300 W, pressure 10 Pa, O_2_ flow rate 50 sccm, and process time 2 min 40 s for the first step; RF power 100 W, pressure 15 Pa, O_2_ flow rate 100 sccm, and process time 1 min for the second step. Prior to releasing the graphene channel, an O_2_ plasma ashing process (power 500 W, pressure 15 Pa, O_2_ flow rate 200 sccm, process time 3 min) was performed to ensure that no residues were left on the surface of the electrodes, ensuring optimal contact of the ionic liquids with the source, drain, and gate electrodes.

### 2.3. Fabrication Process Using LDW-N010

**Figure 3:**
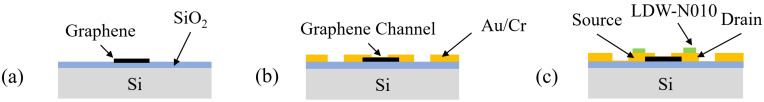
Fabrication process flow using LDW-N010: (**a**) graphene etching by O_2_ plasma; (**b**) Au/Cr electrode deposition by sputter and patterning by lift-off; (**c**) LDW-N010 coating, photolithography, and development.

LDW-N010 offers similar hydrophobic properties to CYTOP^®^ with better process compatibility, but at a much thinner layer, only a few tens of nanometers, which facilitates process integration with less contamination. Another attractive property of LDW-N010 is its photosensitivity and good chemical resistance to ionic liquids. Figure 3 illustrates the detailed process flow when using LDW-N010 to create a hydrophobic ‘ring’-shaped pattern surrounding the graphene channels. The first and second processes were the same as those used in CYTOP^®^. Then, the wafer surface was coated with LDW-N010 via spin-coating (1000 rpm, 60 s). After pre-baking the LDW-N010 at 100 °C for 60 s, exposing it at 300 mJ/cm², pre-baking again at 100 °C for 60 s, and developing it in TMAH for 60 s, the hydrophobic LDW-N010 “ring”-shaped pattern was obtained, as shown in Figure 3c.

The use of LDW-N010 eliminates the need for the oxygen plasma RIE of the CYTOP^®^, as seen in Figure 2d. Consequently, the graphene channels can be released simultaneously using the lift-off process, depicted in Figure 3b, thereby omitting the Au/Cr wet etching process, as shown in Figure 2e. The process depicted in Figure 3 simplified the processes and eliminated the need for high-temperature baking and oxygen plasma RIE of the CYTOP^®^, thereby increasing the process compatibility and yields.

A hydrophobic negative photoresist (ZPN1150-90, ZEON Crop., Tokyo Japan) was used in this work for comparison with the results of using the CYTOP^®^ and the LDW-N010, as well as to investigate the thickness effects of the hydrophobic pattern. The ZPN1150-90 was spin-coated at 3000 rpm for 60 s to achieve a thickness similar to that of the CYTOP^®^.

### 2.4. Device Evaluation

The wettability of the hydrophobic patterns was evaluated using a contact angle meter (PCA11, Kyowa Interface Science Co., Ltd., Niiza, Japan). The thickness of the hydrophobic patterns was measured using a wafer surface profiler (Tencor P-16, KLA Corp., Milpitas, CA, USA). Since the graphene is easily removed by the electron beam when using a scanning electron microscope, the geometry of the fabricated devices was observed using a scanning electron microscope (S-3000H, Hitachi Co., Ltd., Tokyo, Japan), and the graphene channel of the fabricated devices was observed by using a 3D laser scanning microscope (VK-X3000, Keyence Crop., Osaka, Japan). The ionic liquid filling was evaluated using a pipette with a controllable volume range of 100 nL to 2 μL (Nichipet EXII, Nichiryo Co., Ltd., Koshigaya, Japan) and then observed using the 3D laser scanning microscope.

A soil simulation system was developed to investigate the electrical characteristics of the device and its functionalities as a gas sensor. The self-developed soil simulation system includes a sealed chamber with a size of 300 mm × 300 mm × 170 mm, a vaporizer using a heater and a Peltier to artificially mix the target gas components, an electrical fan to circulate the target molecular gas inside the chamber for better reliability and repeatability, and a signal processing module with a source measure unit (Keithley 2450, Tektronix, Inc., Beaverton, OR, USA). To confirm the response of the device and their characteristics for NO_3_-N detection, the fabricated device was set inside the self-developed soil simulation system after the filling of the ionic liquids, and then the current-voltage characteristics of the source-drain electrodes (I_ds_-V_ds_) of the device was measured and recorded. The working principles and the design of the soil simulation system have been published elsewhere [15,16]. More detailed information about the self-developed soil simulation system will be presented, together with our device evaluation results by using various gases, in our future publications.

## 3. Results and Discussion

### 3.1. Microscopic Images of the Fabricated FETs and the Graphene Channel

**Figure 4:**
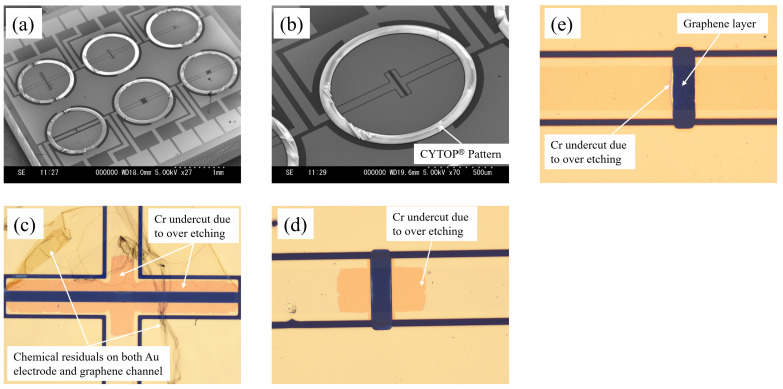
(**a**) SEM image of a fabricated chip using CYTOP^®^; (**b**) SEM image of an individual FET using CYTOP^®^ (length: 20 μm; width: 250 μm); (**c**) optical image of the graphene channel (length: 20 μm; width: 500 μm) with chemical residuals and Cr undercut by wet etching; (**d**) optical image of the graphene channel (length: 20 μm; width: 50 μm) with Cr undercut by wet etching; (**e**) optical image of the graphene channel (length: 20 μm; width: 50 μm) without chemical residuals, the Cr undercut is neglectable.

Figure 4 shows the SEM and optical images of a fabricated chip with 6-channel ionic liquid-gated graphene FETs. A hydrophobic CYTOP^®^ pattern was successfully created surrounding each channel with an inner diameter of 1 mm and a width of 100 μm. The thickness of the CYTOP^®^ was measured as 2782 nm.

To avoid damage to the graphene layer from the oxygen plasma RIE of the CYTOP^®^, the graphene channel was covered by a thin Au/Cr electrode layer until the final step of channel release by wet etching of the Au/Cr, as shown in Figure 2e. However, as shown in Figure 4c, chemical residues were found covering parts of the graphene channel in some FETs, particularly those with larger channel sizes. No chemical residues were found in those FETs with small channel sizes, as shown in Figure 4d.

The chemical residues are believed to be reaction products between the Au wet etchant (AURUM-302, Kanto Chemical Co., Inc., Tokyo, Japan) and the photoresist (THMR-ip3600, Tokyo Ohka Kogyo Co., Ltd., Kawasaki, Japan). Figure 4c,d also reveal that these residues are difficult to remove even using photoresist remover, additional Au wet etching, or additional Cr wet etching, which leads to Cr undercut above the graphene pattern. Kunc J. et al. reported a similar phenomenon during the wet etching of Au on graphene film. They concluded that aqua regia is a viable method for achieving high-quality, resist-free graphene surfaces [23]. In our work, after Au wet etching, an oxygen plasma ashing process was added prior to Cr wet etching to remove the chemical residues. As shown in Figure 4e, no chemical residues were found in the graphene channel. Moreover, the Cr undercut was neglectable and limited at the edge of the Au/Cr electrode layer.

It has been reported that introducing Cr atoms into graphene as a dopant may convert graphene into a magnetic and semiconducting material. This suggests that Cr-doped graphene can be used as a building block for potential electronic devices and as a method for constructing them [24]. Consequently, the FET characteristics of the device may change due to the Cr sputtering on top of the graphene channel, as shown in Figure 2b, and the incomplete Cr wet etching, as shown in Figure 4c. Further research is needed to investigate the effects of Cr sputter and Cr wet etching on graphene channels if the process in Figure 2 is applied for device fabrication.

**Figure 5:**
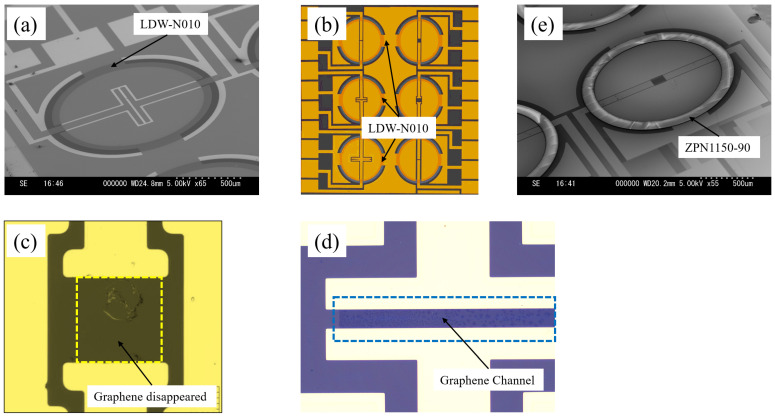
(**a**) SEM image of a fabricated individual FET (length: 20 μm; width: 500 μm) using LDW-N010; (**b**) optical image of the chip with well-defined hydrophobic pattern by LDW-N010; (**c**) optical image of the graphene channel (length: 100 μm; width: 100 μm) after using N-methyl pyrrolidone (NMP) to remove the photoresist during Au/Cr lift-off; (**d**) optical image of the graphene channel (length: 20 μm; width: 200 μm) after using acetone to remove the photoresist during Au/Cr lift-off; (**e**) SEM image of a fabricated individual FET (channel length: 100 μm; width: 50 μm) using negative photoresist ZPN1150-90.

Figure 5 shows the SEM and optical images of a fabricated chip with 6-channel ionic liquid-gated graphene FETs, utilizing the photosensitive hydrophobic layer LDW-N010 to create a hydrophobic pattern surrounding the graphene channel. Although the thickness of the LDW-N010 pattern was measured to be approximately 20 nm, it can be clearly seen from both the SEM image, as shown in Figure 5a, and the optical image, as shown in Figure 5b.

In the process shown in Figure 3, a lift-off technique was used for Au/Cr electrode deposition and patterning, and the graphene channel was covered by a negative photoresist. The use of ultrasonics may help remove Au/Cr and the underlying photoresist, but it is detrimental to the atom-thick graphene layer. Therefore, N-methyl pyrrolidone (NMP, Mitsubishi Chemical Group Corp.) was introduced during the lift-off process as a remover, which unfortunately resulted in fatal damage to the graphene layer, as shown in Figure 5c. Our experiments also demonstrated, as depicted in Figure 5d, that the lift-off of the Au/Cr electrode layer could be successfully achieved using acetone to remove the photoresist and the Au/Cr above the graphene channel, as designed with a minimum channel length of 20 µm.

Figure 5e shows that the graphene FET, which used a negative photoresist ZPN1150-90 to create a hydrophobic ‘ring’-shaped pattern, exhibited a clean graphene channel without any chemical residues. The thickness of the ZPN1150-90 was measured to be 3394 nm.

### 3.2. Ionic Liquids Filling

Table 1 lists the process conditions, measured thickness, and contact angle of the hydrophobic patterns. Although the thickness of the LDW-N010 was much thinner than that of the CYTOP^®^ and the ZPN1150-90, it exhibited a higher contact angle, indicating excellent hydrophobic performance. The contact angle of the CYTOP^®^ was decreased from 113.1° to 78.4° after patterning due to the oxygen plasma treatment prior to photoresist coating.

**Figure 6:**
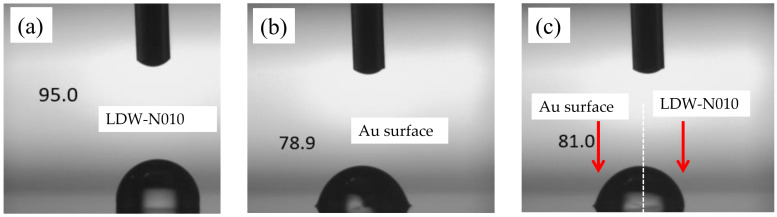
Measured contact angle on (**a**) LDW-N010 surface; (**b**) Au electrode surface without LDW-N010; (**c**) interface surface between Au and LDW-N010.

**Figure 7:**
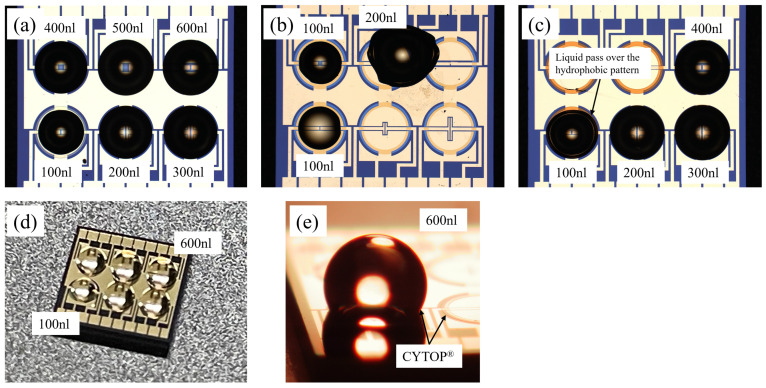
(**a**) optical image of the chip using CYTOP^®^ after filling with H_2_O at various volumes; (**b**) optical image of the chip using LDW-N010 after filling with H_2_O at various volumes; (**c**) optical image of the chip using ZPN1150-90 after filling with H_2_O at various volume; (**d**) photo of the chip using CYTOP^®^ after filling with H_2_O at various volume; (**e**) 3D microscopic image of the chip using CYTOP^®^ and after filling with H_2_O at 600 nL.

Figure 6 illustrates the effectiveness of the hydrophobic LDW-N010 pattern on the Au/Cr electrode through photos of the contact angle. To evaluate the filling process, controlled volumes of liquid H_2_O were used to evaluate the chips developed using the aforementioned processes, with the results shown in Figure 7. A large volume of the ionic liquid is preferred for the pursuit of high sensitivity and selectivity while at the cost of response time. Volumes from 100 nL to 600 nL were used in the filling test to provide more options for various applications.

As seen in Figure 7a,b, the liquids were confined within the hydrophobic ‘ring’-shaped pattern at a volume of 100 nL when using the 2782 nm-thick CYTOP^®^ and the ultra-thin LDW-N010. At a volume of 200 nL, the liquids could not be restricted to the designated area when using LDW-N010, whereas CYTOP^®^ managed to contain the liquid effectively up to 600 nL.

Figure 7d,e indicate that the liquids have not passed through the CYTOP^®^ hydrophobic barriers even at the volume of 600 nL, and none of the liquid was found to have moved over the nearby electrodes because of the combined effects of the surface tension of the H_2_O and the hydrophobic properties of CYTOP^®^. This makes the process potentially suitable for most applications. It is worth noting in Figure 7c that although the liquids were successfully contained up to 400 nL or more by ZPN1150-90, similar to CYTOP^®^ due to the thickness effects and comparable hydrophobic performance, they pass through the ZPN1150-90 hydrophobic barriers at a volume of 100 nL. Additionally, ZPN1150-90 may not tolerate long-term use, particularly with organic solvents, due to its low chemical stability.

The results in Figure 4, Figure 5, Figure 6 and Figure 7 indicated that the use of the ultra-thin photosensitive hydrophobic layer might simplify the device fabrication process from 4-mask to 3-mask and avoid chemical residuals on the surface of the graphene channel while enabling successful filling of liquids with volumes of 100 nL or less. Whereas CYTOP^®^ allows for larger volumes of ionic liquids but at the cost of reduced yields due to chemical residuals on larger channels, additional ashing processes, etc. Generally speaking, a low volume of ionic liquid facilitates faster FET response time, while a larger volume may improve its resolution and sensitivity. Further evaluation using various practical available ionic liquids at different volumes is ongoing to validate these results in practical applications.

### 3.3. Electrical Characteristics of the FETs

**Figure 8:**
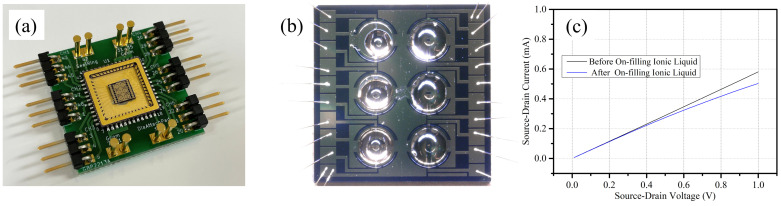
(**a**) Photo of our evaluation module after wire bonding of the ionic liquid-gated graphene FET device onto a ceramic package; (**b**) photo of the chip using CYTOP^®^ after filling ionic liquid [PMIM][BF4] with various volumes; (**c**) current-voltage characteristics of the source-drain electrodes (I_ds_-V_ds_) of the graphene FET before and after filling of the ionic liquid [PMIM][BF4]. No gate voltage was applied during the Ids-Vds test.

As shown in Figure 8a, an evaluation module was designed, and the device was assembled into the module by wire bonding onto a ceramic package for further investigation of the electrical characteristics and the evaluation of various ionic liquids. Figure 8b shows a photo of the fabricated graphene FETs after the ionic liquid [PMIM][BF4] was successfully filled onto the restricted location within the hydrophobic ‘ring’-shaped CYTOP^®^ pattern at various volumes. [PMIM][BF4] was selected in this paper because it may absorb nitrate nitrogen. Other ionic liquids will be examined in future work as well. The measured current-voltage characteristics of the source-drain electrodes (I_ds_-V_ds_), shown in Figure 8c, exhibit linear and typical FET characteristics before and after the ionic liquid filling, and the decrease of the I_ds_ due to the ionic liquid can be clearly identified.

Before investigating the response of the fabricated graphene FETs to NO_3_-N vapor and other nitrogen fertilizers, a reliability test of the graphene FETs was carried out using our self-developed soil simulation system. The drain-source current (I_ds_) at different gate voltages (V_g_) was recorded when both chambers were opened to expose the graphene FETs to free air flow and closed to keep the graphene FETs in a steady, unchanging atmosphere. The gate voltage (V_g_) was changed every 3 s, and the drain-source current (I_ds_) was recorded 2 s after applying 50 mV as drain-source voltage (V_ds_) to ensure a reliable measurement.

**Figure 9:**
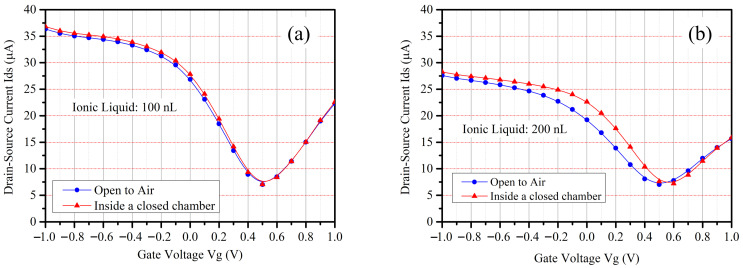
Measure drain-source current (I_ds_) of the graphene FET at different gate voltages (V_g_) when the FET was exposed to air or sealed inside our self-developed soil simulation chamber. The volume of the filled [PMIM][BF4] was (**a**) 100 nL and (**b**) 200 nL, respectively. The measurement was done at 25 °C with a relative humidity of 51.2%.

Figure 9 clearly shows the difference in the drain-source current (I_ds_) between the FETs, which were filled with 100 nL [PMIM][BF4] and 200 nL [PMIM][BF4], suggesting that the volume of the ionic liquid needs to be optimized for a certain application. In addition, Figure 9 also indicates the difference of the drain-source current (I_ds_) when the device was set in open air and inside a closed chamber, suggesting that flowing air and static air may lead to different absorption of gas molecules into the ionic liquid due to pressure variations. The results in Figure 9 provide important references when evaluating and calibrating the device in our self-developed chamber.

The change in drain-source current (I_ds_) of the fabricated graphene FETs in the presence of NO_3_-N vapor has been investigated and confirmed using the soil simulation system. Detailed device performance metrics, including response time dependence, gas selectivity, and others, have been presented elsewhere [16]. Further research results will be presented in our future publications.

## 4. Conclusions

This paper presented the design and fabrication process optimization of a compact, multi-channel ionic liquid-gated graphene FET for gas sensing in smart agriculture. It was demonstrated that the use of an ultra-thin photosensitive hydrophobic layer, LDW-N010, enables a successful filling of ionic liquids with volumes of up to 100 nL and simplified process flow using three photomasks, thus preferred for the pursuit of high process yields. The use of the CYTOP^®^ allows for larger volumes of ionic liquids of over 600 nL but at the cost of reduced yields due to chemical residuals on larger channels, baking during CYTOP^®^ deposition, and potentially Cr-doping effect by direct Cr sputter on the surface of the graphene layer. The preliminary evaluation results show typical FET characteristics of those fabricated devices, which are believed to be applicable to various application environments in smart agriculture.

The devices that have been developed will be further evaluated using our self-developed soil simulation system. The composition and volume of ionic liquids will be optimized to achieve the desired sensitivity while maintaining a fast enough response time for real-time on-site monitoring of NO_3_-N vapor and other nitrogen fertilizers. In addition, the effects of Cr-doping may require fundamental research and dedicated experiments to be fully understood. Further evaluation is ongoing as well to understand the device’s performance in various practical applications.

## Figures and Tables

**Table 1:** Process conditions, thickness, and contact angle of the hydrophobic pattern.

	Coating	Max. Baking Temperature	Patterning	Measured Thickness	Measured Contact Angle	
					As deposited	After Patterning
CYTOP^®^	600 rpm/60 s	200 °C/30 min	O_2_ plasma	2782 nm	113.1°	78.4°
LDW-N010	1000 rpm/60 s	100 °C/60 s	lithography	~20 nm	103.8°	95°
ZPN1150-90	3000 rpm/60 s	110 °C/60 s	lithography	3394 nm		80.5°

## Data Availability

Data are contained within the article.
